# GC-MS/MS Method for Determination of Polycyclic Aromatic Hydrocarbons in Herbal Medicines

**DOI:** 10.3390/molecules28093853

**Published:** 2023-05-02

**Authors:** Jwahaeng Park, Kyuyeob Kim, Dayoun Ryu, Jin-Hee Whang, Jae-Hyung Mah

**Affiliations:** 1 Herbal Medicine Research Division, Ministry of Food and Drug Safety, Cheongju 28159, Republic of Korea; antiss@korea.kr (J.P.); yeop007@korea.kr (K.K.);; 2 Department of Biotechnology, Korea University, Seoul 02841, Republic of Korea; 3 Department of Food and Biotechnology, Korea University, Sejong 30019, Republic of Korea

**Keywords:** GC-MS/MS, polycyclic aromatic hydrocarbon, herbal medicine, validation, monitoring

## Abstract

Polycyclic aromatic hydrocarbons (PAHs) are hydrophobic organic contaminants that have a highly carcinogenic and mutagenic nature. This study aimed to develop and validate a sensitive analytical method to determine 8 PAHs in 51 herbal medicines (HMs) using gas chromatography (GC)-tandem mass spectrometry (MS/MS). Liquid––liquid extraction and florisil SPE cartridge purification were basically adopted for pretreatment. For the samples containing essential oil, starch grain, etc., *N,N*-dimethyl formamide/water mixture (9:1, *v*/*v*) was added in the extraction step. The multiple reaction monitoring (MRM) conditions were newly obtained by the infusion of reference solutions of the targeted compounds at a concentration of 100 ng/mL into the GC-MS/MS system used in this study. The 51 items were classified according to whether or not they contained essential oil. Eight PAHs were not detected in 39 (8.3%) of the 459 samples monitored. The total content of 8 PAHs ranged from 0.45 μg/kg in Anemarrhenae Rhizoma to 270.94 μg/kg in Zingiberis Rhizoma. The average content of those ranged from 0.9 μg/kg in Araliae Continentalis Radix to 110.8 μg/kg in Coptidis Rhizoma Preparata cum Vinum. The results of this study prove that the proposed method is useful for determining 8 PAHs in HMs.

## 1. Introduction

Polycyclic aromatic hydrocarbons (PAHs) are hydrophobic organic contaminants that are composed of two or more fused aromatic rings [1]. Once formed by incomplete combustion, PAHs spread to all environmental components such as air, water, sediment, soil and plants, polluting aquatic and terrestrial species [2]. In 1984, the United States Environmental Protection Agency (USEPA) designated 16 PAHs as priority environmental pollutants of carcinogenic or mutagenic [3]. In 2007, the European Food Safety Authority (EFSA) reported descriptive statistics for the concentrations (μg/kg) of up to 16 PAHs in 9714 food products [4]. According to the report, 3086 samples (31.8%) did not contain any PAH above the limit of detection. In 2008, the EFSA concluded that benzo[a]pyrene (BaP) is not a suitable indicator for the presence of PAHs in food and that both 8 PAHs (benzo[a]anthracene (BaA), chrysene(CHR), benzo[b]fluoranthene (BbF), benzo[k]fluoranthene (BkF), BaP, dibenzo[a,h]anthracene (DahA), benzo[g,h,i]perylene (BghiP) and indeno [1,2,3-c,d]pyrene (IcdP)) and 4 PAHs (BaA, CHR, BbF and BaP) are the most suitable markers of PAHs in food [5,6,7]. The International Agency for Research on Cancer (IARC) classified BaP as Group 1, DahA as Group 2A, BaA, CHR, BbF, BkF and IcdP as Group 2B and BghiP as Group 3 [8].

Since ancient times, herbal medicines (HMs) have been used for various purposes such as food ingredients or therapeutic treatments. In the modern era, although synthetic drugs are effective for various diseases, the interest in substances with the therapeutic effects of plants grown in nature is increasing. However, HMs are facing stumbling blocks like pesticides, heavy metals and PAHs, which are environmental pollutants due to industrialization and civilization, while their use is increasing [9]. In addition to these external factors, the internal PAHs may be generated during the frying, roasting, steaming and smoking processes because some processed herbal medicinal products require long-term cooking at high temperatures [10]. Thus, the European Commission (EC) considered the maximum allowable residual content of Bap to be less than 10.0 μg/kg and the total 4 PAHs to be less than 50 μg/kg in dried herbs [11]. In 2013, the Ministry of Food and Drug Safety of the Republic of Korea (MFDS of Korea) investigated 50 samples of 16 HMs and found that in 6 samples (unpublished data), it exceeded maximum levels (MLs) of 5.0 μg/kg for BaP in Rehmanniae Radix Preparata and Rehmanniae Radix, respectively, notified in the Korean phamacopoeia [12].

Analyses of PAHs have been conducted in many areas, such as air [13], soil [14,15], sediment [16], sludgy [17], river and wastewater [18,19,20], smoke products [21,22], cosmetic products [23] and foods [24,25,26,27,28,29]. Recently, various analytical methods of PAHs for herbal medicines characterized by diverse matrices have been reported as follows. A GC-MS/MS analysis of 16 PAHs combined with pretreatment steps such as liquid–liquid extraction, gel permeation chromatography (GPC) and solid phase extraction (SPE) was reported [30]. The disadvantages of this technique are long run time and high solvent consumption [31]. To overcome those demerits above, three types (ProElut C_18_, ProElut Florisil and ProElut PSA) of SPE columns coupled to tandem mass spectrometry were suggested, respectively, depending on the sample type: ‘roots and stems’, ‘flowers, fruits and seeds’ and ‘leaves and barks’ [9]. A modular clean-up strategy consisting of ‘silica SPE cartridge’ for regular spices, ‘florisil SPE cartridge’ for highly pigmented spices/herbs and ‘silica SPE cartridge following SupelMIP SPE cartridge’ for complex spices has been reported [31]. An effective alternative QuEChERs pretreatment [32] and Magnetic C_60_ SPE purification [33] have also been suggested.

In the compendium of the Korean pharmacopoeia, BaP is currently analyzed using liquid–liquid extraction and SPE column purification connected with a high-performance liquid chromatography (HPLC) fluorescence detector (FLD) [12]. However, there are many limitations in applying the present method for the identification and quantification of different PAHs in diverse HMs because of matrix-originated inhibition factors such as essential oils. To develop a better analytical method in this study, therefore, herbal medicine samples were divided mainly depending on whether or not they contained essential oil. Basically, liquid–liquid extraction and florisil SPE cartridge purification were adopted for pretreatment. As for the samples categorized as containing essential oil, starch grain, etc., *N,N*-dimethyl formamide/water mixture (9:1, *v*/*v*) solution was added in the extraction step to remove inhibition factors. Taken together, based on the application of these techniques, this study modified the pretreatment method according to the properties and characteristics of individual herbal medicine samples and optimized the gas chromatography (GC)-tandem mass spectrometry (MS/MS) instrumental method to determine 8 PAHs in 51 kinds of herbal medicine matrices. The distribution of 8 PAHs in 459 samples of 51 kinds of HMs was also monitored using the developed analytical method.

## 2. Results and Discussion

### 2.1. Optimization of the Analytical Method and Selection of HMs Representatives

**Figure 1:**
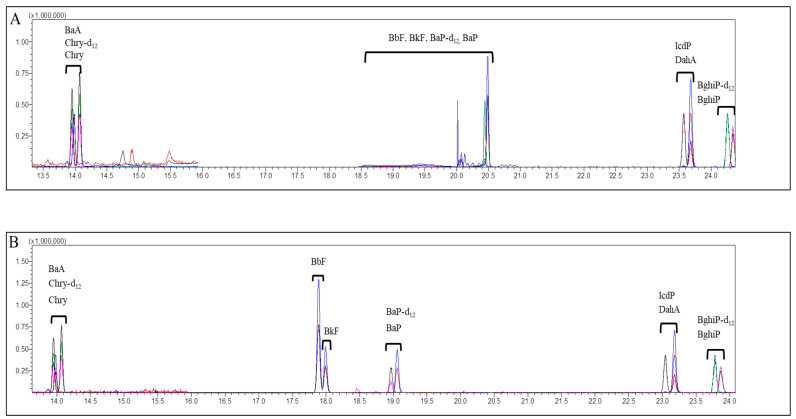
Typical chromatograms of 8 polycyclic aromatic hydrocarbons extracted from Angelicae Gigantis Radix by non-DFA extraction method only using hexane for extraction (**A**) and DFA extraction method (**B**). BaA: benzo[a]anthracene, Chry-d12: internal standard of chrysene, Chry: chrysene, Bbf: benzo[b]fluoranthene, Bkf: benzo[k]fluoranthene, Bap-d12: internal standard of benzo[a]pyrene, Bap: benzo[a]pyrene, IcdP: indeno[1,2,3-c,d]pyrene, DahA: dibenzo[a,h]anthracene, BghiP-d12: internal standard of benzo[g,h,i]perylene, BghiP: benzo[g,h,i]perylene. The x-axis and y-axis represent the retention time (min) and detector signal intensity, respectively.

The extraction method using only hexane has limitations in the quantitative analysis of herbal samples composed of various matrices. In the preliminary test, some target materials could not be ionized well due to the inclusion of essential oil components, resulting in poor peak separation. Such samples had to be subjected to an extraction process in which *N,N*-dimethyl formamide was added, and the hexane layer was extracted again (DFA extraction method) to minimize the influence of the herbal matrix (Figure 1). Still, in the case of some samples containing essential oils, there were some items that had good peaks and recoveries without interfering substances even when extracted only with hexane (non-DFA extraction method), and the DFA extraction method was not required for them. Meanwhile, the MRM conditions for 8 PAHs were set as compiled in Table 1 and were slightly different from those in the study of Anderson et al. [34] in terms of the *m/z* values. The determined MRM mode was found to have high sensitivity for the 8 PAHs in the GC-MS/MS analysis. Consequently, this study set up well-suited quantitative ions and confirmation ions in the MRM mode for the identification and quantification of PAHs in the HMs.

**Figure 2:**
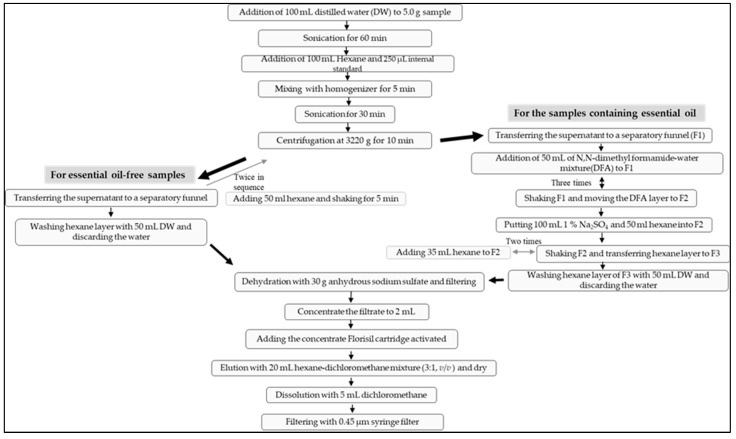
Flow chart of sample extraction and purification.

**Figure 3:**
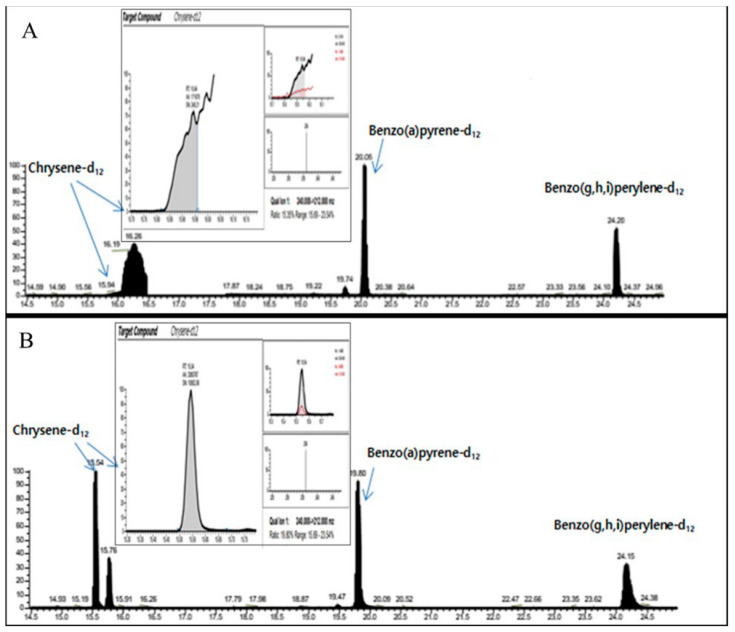
Comparison of the chromatogram for chrysene-d12 obtained by non-DFA extraction (**A**) and DFA extraction (**B**) employed in the pretreatment processing to separate 8 polycyclic aromatic hydrocarbons from Araliae Continentalis Radix. The x-axis and y-axis represent the retention time (min) and detector signal intensity, respectively.

Sanguisorbae Radix Carbonisatum, which showed stable chromatogram baseline and relative low detected concentrations of 8 PAHs, was selected as a representative of the first group of 19 thermal processed HMs. Moreover, all of the first groups showed good peaks in the circumstance of the non-DFA extraction method. The second group, consisting of 13 natural HMs, was classified according to whether or not they contained essential oil, starch grain and calcium oxalate by referring to the properties and characteristics described in the Korean pharmacopoeia. Although Puerariae Radix, Glycyrrhizae Radix et Rhizoma, Atractylodis Rhizoma Alba, Bupleuri Radix, Achyranthis Radix, Ginseng Radix, Cnidii Rhizoma and Cyperi Rhizoma contained essential oil and starch grain, they showed good peaks even by the non-DFA extraction method. So, only Angelicae Gigantis Radix, which has starch granules in its oil cell, was chosen as a separate representative to which the DFA extraction method was applied. An important reason for this choice was that, for instance, Angelicae Gigantis Radix could not be ionized when pretreated by using the non-DFA extraction method (Figure 2). By comparing the blanks of the second group, Achyranthis Radix and Scutellariae Radix, along with Angelicae Gigantis Radix, were all selected as representatives because they exhibited stable chromatogram baselines and relatively low detected concentrations of 8 PAHs. For the third group, consisting of 19 natural HMs, the DFA extraction method was applied for Asiasari Radix et Rhizoma, Acori Graminei Rhizoma, Atractylodis Rhizoma and Araliae Continentalis Radix, which contain essential oil because of the properties and characteristics described in the Korean pharmacopoeia. For example, Araliae Continentalis Radix showed a poor peak for CHR-12 when extracted with the non-DFA extraction method but exhibited a good peak after applying the DFA extraction method (Figure 3). By comparing the blanks of the third group, Asiasari Radix et Rhizoma and Anemarrhenae Rhizoma among 19 HMs of the third group showing stable baselines and relatively low detected concentrations of 8 PAHs, were selected as representatives. Consequently, as for six representatives, the validation factors such as selectivity, linearity, LOD, LOQ and recovery rates were examined and illustrated in following sections.

### 2.2. Matrix Effect

Depending on the matrix of the blank sample, the ionization efficiency can be lower or higher while mass spectrometry is being used [35]. Since most HMs originate from natural plants containing various compounds, which may affect the quantitative measurements, matrix effects are inevitable when analyzing substances or contaminants they contain. Therefore, there is a strong matrix effect that causes a difference in ionization efficiency. To reduce the matrix effect, a calibration curve is prepared using the matrix-matched method in which a standard product is dissolved in a blank sample. That is, when there is a matrix effect, linearity should be evaluated using a standard solution containing the sample matrix [36]. Matrix effect can be classified as strong matrix effect below 50% or above 150%, moderate matrix effect between 50–80% or 120–150%, and low matrix effect between 80–120% [37]. Taking the criteria into account, the matrix effect on 8 PAHs was investigated. The matrix effects for the six representatives are summarized in Table 2. In Sanguisorbae Radix Carbonisatum, although IcdP and DahA had strong matrix effects of 189% and 167%, respectively, those showed acceptable recoveries. Except for IcdP and DahA of Aconiti Ciliare Tuber Preparata and Morindae Radix Preparata cum Vinum, most of the other items showed low to moderate matrix effects on all PAHs (Table S1). It is also noteworthy that, owing to acceptable recoveries that were shown for most of the items, calibration curves could be determined to be prepared by dissolving a standard internal material in a standard solution instead of using the matrix-matched method.

### 2.3. Linearity and Sensitivity

**Figure 4:**
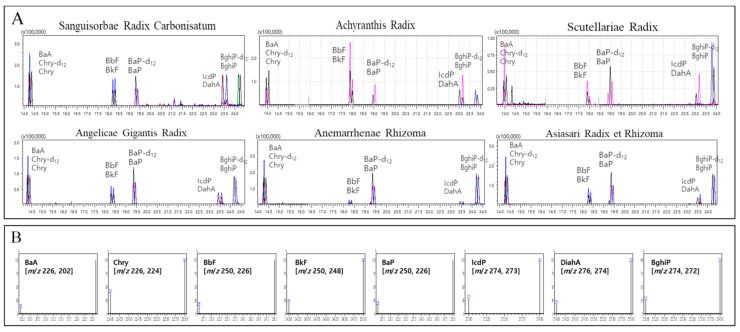
Chromatograms of 6 representative herbal medicines spiked with mixed standard solutions (**A**) and MRM conditions for 8 polycyclic aromatic hydrocarbons (**B**). BaA: benzo[a]anthracene, Chry-d12: internal standard of chrysene, Chry: chrysene, Bbf: benzo[b]fluoranthene, Bkf: benzo[k]fluoranthene, Bap-d12: internal standard of benzo[a]pyrene, Bap: benzo[a]pyrene, IcdP: indeno[1,2,3-c,d]pyrene, DahA: dibenzo[a,h]anthracene, BghiP-d12: internal standard of benzo[g,h,i]perylene, BghiP: benzo[g,h,i]perylene.

The linearity between the analytical concentration and the chromatogram peak was evaluated by the correlation coefficient (R^2^). The R^2^ values for 8 PAHs in the quantitative range (1–100 ng/mL) were greater than 0.998 (Table 1), indicating that the analytical method proposed in this study provided good linearity due to its good selectivity (Figure 4). LOD and LOQ for the 8 PAHs in each of the six representatives were summarized in Table 2. The LOQs of the 8 PAHs were 0.82–1.10 μg/kg for Sanguisorbae Radix Carbonisatum, Asiasari Radix et Rhizoma and Anemarrhenae Rhizoma, 0.35–1.04 μg/kg for Achyranthis Radix, 0.43–1.11 μg/kg for Scutellariae Radix and 0.26 to 1.09 μg/kg for Angelicae Gigantis Radix. Based on the results, the derived GC-MS/MS analysis showed sufficient sensitivity in determining the levels of 8 PAHs in HMs.

### 2.4. Recovery and Precision

Standard substances corresponding to three different concentrations (low, medium, and high), such as 2, 5, and 10 times the LOQ, were added to the samples within the quantification range of six representatives. The accuracy of 8 PAHs was evaluated with the recovery rate calculated from the measured results. The precision was evaluated as the relative standard deviation (RSD) of the measured results. The recovery rates and RSDs are described in Table 2. As for Sanguisorbae Radix Carbonisatum, the recovery rate of PAHs was 66.4–104.7%, with the intra-day RSD of 0.8–22.3% and the inter-day RSDr of 1.0–17.1%. The recovery rate from Achyranthis Radix was 71.2–106.5%, with the intra-day RSD of 0.5–9.8% and the inter-day RSDr of 0.5–15.1%. The recovery rate from Scutellariae Radix was 54.7–125.0%, with the intra-day RSD of 0.4–12.3% and the inter-day RSDr of 3.1–32.1%. The recovery rate from Angelicae Gigantis Radix was 70.7–94.3%, with the intra-day RSD of 0.6–14.3% and the inter-day RSDr of 1.1–22.9%. The recovery rate from Anemarrhenae Rhizoma was 83.7–124.4%, with the intra-day RSD of 1.2–13.7% and the inter-day RSDr of 0.3–17.2%. The recovery rate from Asiasari Radix et Rhizoma was 70.2–122.2%, with the intra-day RSD of 1.5–19.0% and the inter-day RSDr of 1.0–25.1%. Interestingly, both the minimum and maximum values of recovery rates were found in Scutellariae Radix among the six representatives. In detail, the highest recovery rate was 125.0% at 2 LOQ for BaA, and the lowest recovery rate was 54.7% at 10 LOQ for CHR (Table S2). It is speculated that this is probably due to the characteristic matrix of Scutellariae Radix. In the near future, analytical studies on the differences in matrices of HMs by nutritional analysts are required.

As stated above, the validation factors obtained through these verified methods conformed to the AOAC guidelines [38]. Particularly, the recoveries of PAHs at low, medium and high concentrations appeared to be within an acceptable range, although Scutellariae Radix, Anemarrhenae Rhizoma and Asiasari Radix et Rhizoma showed slightly overestimated recovery values. In addition, the RSDs were entirely reasonable and satisfactory values.

### 2.5. Cross Validation

A cross-validation experiment was performed to validate the proposed analytical method by comparatively evaluating inter-laboratory selectivity, linearity, LOD, LOQ, recovery and precision. For this purpose, three different concentrations (low, medium and high concentrations set as 2, 5 and 10 times the LOQ, respectively) of standard substances were added to the six representatives. As a result, no disturbed peaks appeared in the chromatogram of GC-MS/MS. In all cases, the linearity was greater than 0.994, and the LOQs for the 8 PAHs in each of the six representatives were in the range of 0.44 and 1.18 µg/kg (Table 3). The recoveries of Sanguisorbae Radix Carbonisatum, Achyranthis Radix, Scutellariae Radix, Angelicae Gigantis Radix, Anemarrhenae Rhizoma and Asiasari Radix et Rhizoma were 75.3–107.1%, 70.4–123.6%, 74.0–123.7%, 71.3–121.7%, 73.0–105.5% and 78.5–118.0%, respectively, and their RSDs were 0.2–6.3%, 0.2–5.5%, 0.3–6.3%, 0.2–7.2%, 0.5–6.8% and 0.2–8.6%, respectively (Table 3). Since the recoveries and RSDs of most samples obtained through cross-validation satisfied the acceptance criteria of AOAC [38], the proposed analytical method was considered to be useful for determining 8 PAHs in six representatives.

### 2.6. Applicability

This newly developed method was further tested by applying it to a total of 45 items, excluding the six representatives analyzed during the validation process presented in this study. The recoveries were tested at two times the LOQ, whereas Linderae Radix (BbF, BkF, BaP) and Atractylodis Rhizoma (IcdP, DahA) were specifically tested at five times the LOQ. The recoveries and RSDs averaged 86.7 ± 15.1% and 8.5 ± 6.9%, respectively (Table S3). Therefore, this analytical method is considered to have good applicability based on good recoveries and precision shown in most samples, although the RSDs of BaP in Zingiberis Rhizoma and Aconiti Lateralis Radix Preparata were as high as 54.9% and 24.6%, respectively.

### 2.7. Monitoring of PAHs in Herbal Medicines

The newly developed GC-MS/MS method was applied to survey the pollution level of 8 PAHs in HMs purchased from the pharmaceutical markets in Korea. In this monitoring study, 459 samples of 51 different medicinal herbs were used. As a result, in only 8.3% (39 of 459 samples) and 37.3% (19 of 51 items), none of the 8 PAHs were detected above LOQs. Excluding samples in which all PAHs were not detected, the sum of 8 PAHs in individual 51 HMs ranged from 0.45 μg/kg in Anemarrhenae Rhizoma to 270.94 μg/kg in Zingiberis Rhizoma, with the average content ranging from 0.9 μg/kg in Araliae Continentalis Radix to 110.8 μg/kg in Coptidis Rhizoma Preparata cum Vinum. In terms of risk potential, it is worth mentioning that the sum of 8 PAHs exceeded the maximum allowable total amount of 4 PAHs of 50 μg/kg proposed by the EC [11] in 7.8% (36 samples) of the 459 samples and 37.3% (19 items) of the 51 kinds of HMs, although EFSA concluded that a system of eight substances (8 PAHs) would not provide much added value compared to a system of four substances (4 PAHs) [5]. In particular, 9 out of 19 items polluted by 8 PAHs above the LOQs belonged to the first group consisting of thermally processed herbal medicinal products (Table S4). Such PAHs might be inferred to be originated from the production process of those products. In addition, CHR was detected at 122.87 μg/kg in Aucklandiae Radix, which was the highest level among all the polluted levels of 8 PAHs.

In another study [39], a risk assessment was conducted utilizing information related to the average amount of 8 PAHs, dietary exposure and benchmark dose lower confidence limit (BMDL_10_) based on the contamination level of the 8 PAHs in 51 HMs. HMs are usually available as decoctions or pills. Therefore, in that study, when consumed as a decoction, the average and highest conversion rates from ingredients to decoction were considered to be 6% and 20%, respectively. In the case of pills, a conversion rate of 100% was applied, assuming that the entire amount of pills was filled with HMs. As a result, by calculating the BMDL_10_ (0.49 mg/kg body weight/day), daily exposure and dietary exposure (assuming the conversion rates of decoction and pill to be 20% and 100%, respectively), the margin of exposure (MOE) was found to be 10^5^ or higher. According to the Food Standards Agency, United Kingdom [40], MOEs between 10^4^–10^6^ were considered ‘unlikely to be of concern’, and those above 10^6^ were considered ‘highly unlikely to be of concern’. Therefore, although the potential harm of PAHs in HMs to Koreans could be considered low or negligible, action to minimize future exposure is still needed.

## 3. Materials and Methods

### 3.1. Chemicals and Reagents

Eight standards and two internal standards used in this study were purchased from Sigma-Aldrich (St. Louis, MO, USA). Another internal standard (BghiP-d12) was bought from Cambridge Isotope Laboratories (Wesel, Germany). Their abbreviations are shown in Table 1. HPLC-graded ethanol, hexane, dichloromethane and acetonitrile (ACN) were purchased from Merck KGaA (Darmstadt, Germany). *N,N*-dimethyl formamide of HPLC grade was manufactured by Daejung (Siheung, Korea). Sodium sulfate anhydrous was obtained from Junsei (Tokyo, Japan). LC-MS graded Water was purchased from Sigma-Aldrich (Oakville, ON, Canada). Hexane/dichloromethane mixture (3:1, *v*/*v*) was prepared by mixing 600 mL of hexane and 200 mL of dichloromethane. *N,N*-dimethyl formamide-water mixture (9:1, *v*/*v*) was prepared by mixing 100 mL of water with 900 mL of *N,N*-dimethyl formamide. A 1% sodium sulfate solution was prepared by weighing 10 g of sodium sulfate and dissolving it in 1 L of water.

### 3.2. Standard Solution Preparation

Three kinds of internal standards were used when calibrating 8 PAHs. CHR-d12 for BaA and CHR, BaP-d12 for BbF, BkF and BaP and BghiP-d12 for IcdP, DahA and BghiP were used as the internal standards. After weighing 2 mg of 8 types of standards and 3 types of internal standards individually, they were dissolved in 20 mL of ethanol to prepare 100 μg/mL of individual PAH standard stock solution and internal standard stock solution. The mixed standard solution of 8 PAHs was prepared at 1000 ng/mL and 500 ng/mL by taking a part of the volume of each standard stock solution and mixing them with the dichloromethane. The mixed internal standard solution was prepared at 1000 ng/mL by taking a part of the volume of each stock solution and mixing them with the dichloromethane. Through preliminary tests, the suitable calibration concentrations of 8 PAHs were designed into 1, 2, 5, 10, 25, 50 and 100 ng/mL. The calibration concentration of internal standard was also prepared to be 50 ng/mL. Calibration curves were established using 7 concentrations ranging from 1 to 100 ng/mL according to an internal calibration methodology.

### 3.3. Sample Preparation

Referring to the history of detection of BaP (unpublished data from MFDS), based on the high probability of generation of PAHs during thermal manufacturing process and the top 30% of production rates, 51 kinds of HMs were collected. Due to the efficiency of experiment and space constraints, HMs were divided into three groups. As the first group, 19 thermal processed HMs such as Glycyrrhizae Radix (*Glycyrrhiza uralensis* Fischer) Preparata cum Mel, Glycyrrhizae Radix Preparata, Zingiberis Rhizoma (*Zingiber officinale* Roscoe) Carbonisatum, Eucommiae Cortex (*Eucommia ulmoides* Oliver) Preparata cum Zingiberis Rhizoma Crudus, Eucommiae Cortex Preparata cum Sal, Eucommiae Cortex Carbonisatum, Rhei Rhizoma Preparata cum Vinum (*Rheum palmatum* Linné), Pinelliae Tuber (*Pinellia ternata* Breitenbach) cum Zingiberis Rhizoma Crudus et Alumen (Potassium aluminium sulfate), Psoraleae Semen (*Psoralea corylifolia* Linné) Preparata cum Sal, Evodiae Fructus(*Evodia rutaecarpa* Bentham) Preparata cum Glycyrrhizae Radix, Polygalae Radix (*Polygala tenuifolia* Willdenow) Preparata cum Glycyrrhizae Radix, Polygalae Radix Preparata cum Mel, Sanguisorbae Radix (*Sanguisorba officinalis* Linné) Carbonisatum, Aconiti Ciliare Tuber (*Aconitum kusnezoffii* Reichb) Preparata, Morindae Radix (*Morinda officinalis* How) Preparata cum Vinum, Schizonepetae Spica (*Schizonepeta tenuifolia* Briquet) Carbonisatum, Coptidis Rhizoma (*Coptis japonica* Makino) Preparata cum Vinum, Phellodendri Cortex (*Phellodendron amurense* Ruprecht) Preparata cum Sal and Siegesbeckiae Herba (*Siegesbeckia pubescens* Makino) Preparata cum Vinum were purchased, identified and stored. As the second, 13 natural HMs such as Puerariae Radix (*Pueraria lobata* Ohwi), Glycyrrhizae Radix et Rhizoma (*Glycyrrhiza uralensis* Fischer), Platycodonis Radix (*Platycodon grandiflorum* A. De Candolle), Angelicae Gigantis Radix (*Angelica gigas* Nakai), Atractylodis Rhizoma Alba (*Atractylodes japonica* Koidz), Bupleuri Radix (*Bupleurum falcatum* Linné), Achyranthis Radix (*Achyranthes japonica* Nakai), Ginseng Radix (*Panax ginseng* C. A. Meyer), Paeoniae Radix (*Paeonia lactiflora* Pallas), Cnidii Rhizoma (*Cnidium officinale* Makino), Cyperi Rhizoma (*Cyperus rotundus* Linné), Scutellariae Radix (*Scutellaria baicalensis* Georgi) and Astragali Radix (*Astragalus membranaceus* Bunge) were purchased, identified and stored. As the third, 19 natural HMs such Osterici seu Notopterygii Radix et Rhizoma (*Ostericum koreanum* Maximowicz), Zingiberis Rhizoma (*Zingiber officinale* Roscoe), Araliae Continentalis Radix (*Aralia continentalis* Kitagawa), Liriopis seu Ophiopogonis Tuber (*Liriope platyphylla* Wang et Tang), Aucklandiae Radix (*Aucklandia lappa* Decne), Pinelliae Tuber (*Pinellia ternata* Breitenbach), Saposhnikoviae Radix (*Saposhnikovia divaricata* Schischkin), Angelicae Dahuricae Radix (*Angelica dahurica* Bentham et Hooker f.), Aconiti Lateralis Radix (*Aconitum carmichaeli* Debeaux) Preparata, Dioscoreae Rhizoma (*Dioscorea batatas* Decaisne), Acori Graminei Rhizoma (*Acorus gramineus* Solander), Asiasari Radix et Rhizoma (*Asiasarum heterotropoides* F. Maekawa var.), Linderae Radix (*Lindera strichnifolia* Fernandez-Villar), Clematidis Radix (*Clematis mandshurica* Ruprecht), Anemarrhenae Rhizoma (*Anemarrhena asphodeloides* Bunge), Atractylodis Rhizoma (*Atractylodes lancea* DC.), Gastrodiae Rhizoma (*Gastrodia elata* Blume), Asparagi Tuber (*Asparagus cochinchinensis* Merrill) and Corydalis Tuber (*Corydalis ternata* Nakai) were purchased, identified and stored.

**Figure 5:**
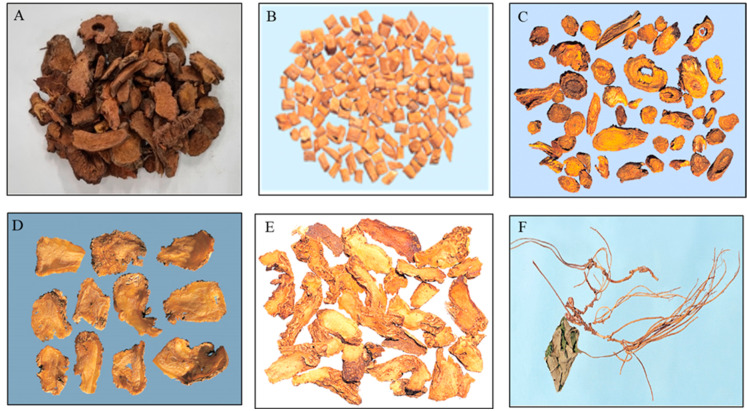
Pictures of 6 kinds of herbal medicines used as representatives in this study. (**A**) Sanguisorbae Radix (*Sanguisorba officinalis* Linne) Carbonisatum, (**B**) Achyranthis Radix (*cutellaria baicalensis* Georgi), (**C**) Scutellariae Radix (*Achyranthes japonica* Nakai), (**D**) Angelicae Gigantis Radix (*Angelica gigas* Nakai), (**E**) Anemarrhenae Rhizoma (*Anemarrhena asphodeloides* Bunge), (**F**) Asiasari Radix et Rhizoma (*Asiasarum heterotropoides* F. Maekawa var.).

For the convenience of experimental process for validation, the samples were represented by 6 kinds of HMs according to appearance, matrix characteristics and stable chromatographic baseline seen in each group. Sanguisorbae Radix Carbonisatum of the first group, Achyranthis Radix, Scutellariae Radix and Angelicae Gigantis Radix of the second and Anemarrhenae Rhizoma and Asiasari Radix et Rhizoma of the last group were chosen as representatives (Figure 5).

A total of 459 samples of 51 HMs, as described above, were purchased from herbal medicinal markets located in Daejeon, Korea, and used in this study. About 70% of samples were produced and imported from China. About 29% of samples were produced in Korea. Before carrying out any further experiments, the identities of the HMs were confirmed through the sensory test by the specialists. Voucher specimens were deposited at the National Institute of Food and Drug Safety Evaluation (Cheongju, Korea). The samples were chopped into small pieces and stored at −18 °C. About 500–600 g of each sample was equally divided into four parts to make them homogeneous. One of the sample parts was pulverized by a grinder (KSP-35, Koreamedi, Daegu, Korea).

### 3.4. Sample Extraction

Liquid–liquid extraction was conducted in two ways depending on whether the samples had essential oil or not. For the essential oil-free samples, *N,N*-dimethyl formamide-water mixture (9:1, *v*/*v*) was not added (hereinafter, referred to as non-DFA extraction method). At the first step, 5.0 g of the ground sample was weighed in 250 mL bottle (Nalgene, Thermo Fisher Scientific^TM^, Waltham, MA, USA). After adding 100 mL of distilled water thereto, sonication was performed for 60 min in an ultrasonic extractor (8510, Branson, St. Louis, MO, USA) at room temperature. Then, 100 mL of hexane and 250 μL of mixed internal standard solution (1000 ng/mL) were added thereto and were mixed with a homogenizer (Mixer 2, OMNI, Atlanta, GA, USA) for 5 min. After sonicating again for 30 min, centrifugation was performed at 3220× *g* for 10 min in a centrifuge (Eppendorf, Hamburg, Germany). The supernatant, the hexane layer, was transferred to a separatory funnel. As for the remains in the bottle, adding 50 mL of hexane, shaking for 5 min in a shaker (Jisco, Seoul, Korea), centrifuging at 3220× *g* and transferring the supernatant to the same separatory funnel were conducted twice in sequence. After combining all the hexane layers obtained above in the separatory funnel, the hexane layer was washed by adding 50 mL of water. After discarding the water, the hexane layer was dehydrated by adding about 30 g of anhydrous sodium sulfate (Waco, Osaka, Japan) and filtered through Whatman No. 41 filter paper (Whatman International Ltd., Buckinghamshire, UK). The filtrate was concentrated to 2 mL under reduced pressure of 250 mbar at 45 °C in a water bath (Heidolph, Schwabach, Germany) (Figure 2).

In the case of the samples containing essential oil, after transferring the supernatant hexane layer to a separatory funnel (F1), the step of adding *N,N*-dimethyl formamide-water mixture (9:1, *v*/*v*) to the separatory funnel was introduced (hereinafter, referred to as DFA extraction method). That is, 50 mL of *N,N*-dimethyl formamide-water mixture (9:1, *v*/*v*) was added to the hexane layer of F1, and after shaking it, the layer of the *N,N*-dimethyl formamide–water mixture was moved to another separatory funnel (F2). This step was repeated three times. After that, 100 mL of 1% sodium sulfate solution (Merck, Darmstadt, Germany) and 50 mL of hexane were put into F2. After shaking, the separated layer of hexane was transferred to the other separatory funnel (F3). Then, 35 mL of hexane was added to F2, shaken, and the hexane layer was also transferred to F3. This step, including adding 35 mL of hexane and transferring from F2 to F3, was repeated once more. Next, the hexane layer of F3 was washed by adding 50 mL of water, and the water was discarded. The remained hexane layer in the F3 was dehydrated by adding about 30 g of anhydrous sodium sulfate and filtered through Whatman No. 41 filter paper. The filtrate was concentrated to 2 mL under reduced pressure of 250 mbar at 45 °C in a water bath (Figure 2).

### 3.5. Sample Purification

In this study, SPE was applied for the purification procedure in basic. The 1g/6cc Florisil cartridge (Waters Oasis, Waters Corp., Milford, MA, USA) was activated by flowing out 10 mL of dichloromethane and 20 mL of hexane at a rate of 2–3 drops per second before use. After adding the concentrate to the activated cartridge, 20 mL of hexane-dichloromethane mixture (3:1, *v*/*v*) was eluted at a rate of 2–3 drops per second. The eluate was blown away under nitrogen gas at 35 °C, and the residue was dissolved again with 5 mL of dichloromethane and filtered through 0.45 μm syringe filter for the instrumental analysis (Figure 2).

### 3.6. GC-MS/MS Analysis

The GC-MS/MS system was coupled to Shimadzu Nexis GC-2030, Shimadzu GCMS-TQ8040NX tandem mass spectrometer in multiple reaction monitoring modes, Shimadzu AOC-20i Plus Auto-Injector and Shimadzu GCMSsolution workstation (Version 4.52) software (Shimadzu, Kyoto, Japan) in which Smart MRM (multiple reaction monitoring) creates MS/MS measurement program automatically. Analytes were separated with Agilent Technologies 30 m × 0.25 mm × 0.25 μm, DB-5MS column. Flow rate was 2 mL/min using helium as a carrier gas. The column oven temperature was programmed as follows: the initial temperature was 40 °C, increased to 220 °C at 30 °C/min, then ramped to 320 °C at 5 °C/min, which was held for 2 min. The total runtime was 28 min. The temperature of the injection port was maintained at 310 °C and injection volume was 1.0 μL in the splitless mode. The temperature of ion source and interface was 250 and 310 °C, respectively. Argon was used as a collision gas (2.0 mL/min).

The MRM for all scan transitions was performed in positive ion mode and at event times of 0.10 to 0.15 sec/cycle. The initial condition in terms of the *m/z* values, mainly referring to the study of Anderson et al. [34], was set by infusing reference solutions at a concentration of 1 mg/L in the scan mode of GC-MS/MS. Including the optimal precursor, ionic products, and collision energy for each PAH, MRM conditions were newly obtained by the infusion of reference solutions of the targeted compounds at a concentration of 100 ng/mL into the GC-MS/MS system. The MRM conditions for 8 PAHs established in this study appear in Table 1.

### 3.7. Statistical Evaluations

All experiments were carried out in triplicate unless stated otherwise. The results given are the average values. Statistical calculations and data analyses were performed using Microsoft Excel (version 2016, Microsoft, Redmond, WA, USA).

### 3.8. Matrix Effect

This study evaluated the matrix effect prior to conducting validation studies. When analysis using a mass spectrometer is performed, the ionization efficiency may be lowered or higher depending on the matrix of the blank sample [35]. Therefore, for each target sample, a standard solution containing a matrix by dissolving the standard in a blank sample and a standard solution in which the standard was dissolved in a solvent were prepared, respectively. Then, the degree of influence of the matrix was evaluated by comparing the slope of the calibration curve between the standard solution containing the matrix and the standard solution dissolved in the solvent. In addition, the blank sample solutions were prepared by the same pretreatment method described above. The calibration curves were achieved from seven-point concentrations ranging from 1 to 100 ng/mL. The matrix effect was evaluated by the following formula [37]: Matrix effect (ME, %) = (slope of the calibration curve in matrix/slope of the calibration curve in solvent) × 100.

### 3.9. Method Validation

Method validation followed the AOAC Official Methods of Analysis guidelines [38]. The validation study was designed by obtaining three different individual samples per matrix. Pulling out selectivity and specificity, linearity, limit of detection (LOD) and limit of quantification (LOQ), and accuracy and precision were performed to verify this study. For selectivity and specificity, the separation and discrimination of analytes in the presence of interfering substances were compared and confirmed by measuring the matrix blank, the PAH standard solution and the mixture of the standard and matrix. For linearity, a calibration curve was prepared with the concentration of the analyte and the peak area ratio by measuring the standard solution of 7 concentrations with an analysis device, and then the correlation coefficient was confirmed. LOD and LOQ were calculated based on the standard deviation of the response (σ) and the slope of the calibration curve (S) by the following equation: LOD = 3.3 × σ / S, LOQ = 10 × σ / S.

For measuring the accuracy and precision, three different levels of standard solution were spiked into blank samples to achieve 2, 5 and 10 times (low, medium and high) the LOQ. After performing pretreatment with the spiked herbal medicine samples, the quantification analysis was conducted. The intra-day precision (repeatability, relative standard deviation [RSD]) and accuracy (recovery rates) were evaluated through the analysis on a single day. Along with these, the inter-day precision (reproducibility, RSDr) and accuracy were derived three times over three consecutive days (*n* = 9). To verify the validity of the analytical method, cross-validation between internal and external laboratories was conducted for selectivity, specificity, linearity, LOD, LOQ, accuracy and precision.

### 3.10. Monitoring of 8 PAHs in Herbal Medicine

Contamination levels of 8 PAHs in HMs were investigated by this modified GC-MS/MS analytical method. A total of 459 samples of 51 items (refer to Sample Preparation section) were collected, identified, stored, pretreated and analyzed for the polluted levels of 8 PAHs.

## 4. Conclusions

The determination method of 8 PAHs in six representatives of HMs was successfully improved by liquid and liquid extraction followed by florisil SPE purification and subsequent GC-MS/MS analysis. In particular, extraction steps were applied depending on whether the individual HMs contained essential oil components. With MRM conditions set for 8 PAHs, the MRM mode showed a high sensitivity for the 8 PAHs in the GC-MS/MS analysis. Meanwhile, by applying this method to monitor the contamination level of 8 PAHs in 459 samples of 51 kinds of HMs purchased from Korean pharmaceutical markets, any PAH above LOQs was detected in 91.7% of all samples. Based on the MOEs from these results, risks attributable to the PAHs in HMs could be interpreted as low or negligible, with action minimizing future exposure. Based on the good applicability, the developed analytical method is expected to be used as a standard analytical method not only for HMs but for agricultural products and foodstuffs that may contain PAHs.

## Figures and Tables

**Table 1:** The chemical information of 8 PAHs used in this study, linearity and MRM conditions established for the GC-MS/MS analysis.

Compounds	Abbreviations	Retention Time (min)	Formula	CAS No.	Linearity (R^2^)	Precursor Ion [M+H]^+^	Product Ion		Collision Energy	
							Target Ion	Reference Ion	Target Ion	Reference Ion
Benz(a)anthracene	BaA	14.263	C_18_H_12_	56-55-3	0.9988	228	226	202	18	27
Chrysene	Chry	14.293	C_18_H_12_	218-01-9	0.9977	228	226	224	33	45
Benzo(b)fluoranthene	BbF	14.385	C_20_H_12_	205-99-2	0.9981	252	250	226	39	27
Benzo(k)fluoranthene	BkF	18.234	C_20_H_12_	207-08-9	0.9984	252	250	248	24	57
Benzo(a)pyrene	BaP	18.34	C_20_H_12_	50-32-8	0.9983	252	250	226	39	24
Indeno(1,2,3-cd)pyrene	IcdP	19.322	C_22_H_12_	193-39-5	0.9985	276	274	273	30	60
Dibenz(a,h)anthracene	DahA	19.413	C_22_H_14_	53-70-3	0.9988	278	276	274	24	60
Benzo(g,h,i)perylene	BghiP	23.392	C_22_H_12_	191-24-2	0.9986	276	274	272	27	60
Chrysene-d12	Chry-d12	23.543	C_18_D_12_	1719-03-5	^1^-	240	236	212	36	24
Benzo(a)pyrene-d12	BaP-d12	24.141	C_20_D_12_	63466-71-7	-	264	260	234	27	36
Benzo(g,h,i)perylene-d12	BghiP-d12	24.231	C_22_D_12_	93951-66-7	-	288	284	286	45	36

^1^ The linearity of each internal standard was not determined.

**Table 2:** The LOD, LOQ, intra- and inter-day recovery and RSDs and matrix effects for 8 PAHs in 6 representative samples.

Sample Items	LOD (µg/kg)	LOQ (µg /kg)	Spiked Concentration (Times of LOQ)	Intra-Day, ^1^ *n* = 9		Inter-Day, *n* = 9		Matrix Effect (%)
				Recovery (%)	^2^ RSD (%)	Recovery (%)	^3^ RSDr (%)	
Sanguisorbae Radix Carbonisatum	0.27–0.36	0.82–1.10	2	75.0–104.7	0.8–22.3	81.9–95.8	3.7–16.4	101–189
			5	66.4–80.9	6.0–13.5	76.4–86.3	2.3–17.1	
			10	69.7–103.9	9.6 –13.6	73.3–100.7	1.0–13.7	
Achyranthis Radix	0.12–0.34	0.35–1.04	2	71.2–106.5	3.1–9.8	77.8–121.0	3.9–15.1	109–132
			5	72.2–88.9	0.5–4.7	72.2–88.6	0.5–9.9	
			10	72.5–80.6	0.6–4.7	70.8–79.7	0.7–4.8	
Scutellariae Radix	0.14–0.37	0.43–1.11	2	76.9–125.0	5.6–9.4	76.2–122.3	5.3–32.1	105–124
			5	66.2–107.9	2.7–12.3	61.7–96.8	8.3–19.0	
			10	54.7–86.6	0.4–2.2	51.7–75.2	3.1–16.6	
Angelicae Gigantis Radix	0.09–0.36	0.26–1.09	2	70.7–94.3	2.3–13.9	74.5–88.4	4.5–22.9	94–106
			5	71.4–93.0	0.6–14.3	77.1–92.1	1.1–14.6	
			10	74.4–85.3	0.8–5.1	76.2–84.8	4.0–6.9	
Anemarrhenae Rhizoma	0.27–0.37	0.82–1.10	2	83.7–105.2	2.7–10.0	87.9–109.8	2.6–17.2	107–117
			5	92.6–105.7	1.6–13.7	96.2–108.2	2.4–11.1	
			10	87.6–110.5	1.2–12.0	94.4–110.2	0.3–7.1	
Asiasari Radix et Rhizoma	0.27–0.37	0.82–1.10	2	70.2–100.1	1.5–19.0	81.4–101.6	1.4–25.1	96–120
			5	75.3–114.4	6.2–13.5	78.6–101.0	1.3–12.1	
			10	73.6–122.2	8.0–10.6	80.3–107.0	1.0–12.6	

^1^ n: number of analyses. ^2^ RSD: intra-day relative standard deviation (repeatability). ^3^ RSDr: inter-day relative standard deviation (reproducibility).

**Table 3:** LOD, LOQ, linearity, intra- and inter-day recovery and RSDs for 8 PAHs in 6 representative samples for cross-validation.

Sample Items	LOD (µg/kg)	LOQ (µg/kg)	Linearity (R^2^)	Intra-Day, ^1^ *n* = 9		Inter-Day, *n* = 9	
				Recovery (%)	^2^ RSD (%)	Recovery (%)	^3^ RSDr (%)
Sanguisorbae Radix Carbonisatum	0.20–0.37	0.61–1.12	0.994–0.999	75.3–107.1	0.2–6.3	77.6–98.8	1.3–18.9
Achyranthis Radix	0.22–0.39	0.67–1.18	0.999–1.000	70.4–123.6	0.2–5.5	73.6–117.3	0.8–3.9
Scutellariae Radix	0.22–0.39	0.67–1.18	0.999–1.000	74.0–123.7	0.3–6.3	73.5–123.4	0.8–3.9
Angelicae Gigantis Radix	0.22–0.39	0.67–1.18	0.999–1.000	71.3–121.7	0.2–7.2	73.1–122.6	0.6–4.6
Anemarrhenae Rhizoma	0.15–0.32	0.44–0.96	0.998–0.999	73.0–105.5	0.5–6.8	75.2–120.3	0.5–3.9
Asiasari Radix et Rhizoma	0.15–0.32	0.44–0.96	0.998–0.999	78.5–118.0.	0.2–8.6	80.7–110.4	0.6–5.5

^1^ n: number of analyses. ^2^ RSD: intra-day relative standard deviation (repeatability). ^3^ RSDr: inter-day relative standard deviation (reproducibility).

## Data Availability

The datasets generated for this study are available on request to the corresponding author.

## Supplementary Materials

The following supporting information can be downloaded at: https://www.mdpi.com/article/10.3390/molecules28093853/s1, Table S1: Matrix effects of 35 kinds of herbal medicines; Table S2: LOD, LOQ, recoveries, intra-day and inter-day RSDs and matrix effects (ME) obtained using the GC-MS/MS in Scutellariae Radix; Table S3: Recoveries and precisions of 45 kinds of herbal medicines for applicability excepting 6 representatives; Table S4: The item lists of the sum of 8 PAHs is over 50 μg/kg among 459 samples of 51 kinds of herbal medicines and maximum concentration in the same items.

## Sample Availability

Samples of the compounds are not available from the authors.

## References

[1] Keyte I.J., Harrison R.M., Lammel G. (2013). Chemical reactivity and long-range transport potential of polycyclic aromatic hydrocarbons—A review. Chem. Soc. Rev..

[2] Paris A., Ledauphin J., Poinot P., Gaillard J.-L. (2018). Polycyclic aromatic hydrocarbons in fruits and vegetables: Origin, analysis, and occurrence. Environ. Pollut..

[3] Veyrand B., Brosseaud A., Sarcher L., Varlet V., Monteau F., Marchand P., Andre F., Le Bizec B. (2007). Innovative method for determination of 19 polycyclic aromatic hydrocarbons in food and oil samples using gas chromatography coupled to tandem mass spectrometry based on an isotope dilution approach. J. Chromatogr. A.

[4] European Food Safety Authority (EFSA) (2007). Findings of the EFSA Data Collection on Polycyclic Aromatic Hydrocarbons in Food. EFSA J..

[5] European Food Safety Authority (2008). Polycyclic Aromatic Hydrocarbons in Food—Scientific Opinion of the Panel on Contaminants in the Food Chain. EFSA J..

[6] Wretling S., Eriksson A., Eskhult G.A., Larsson B. (2010). Polycyclic aromatic hydrocarbons (PAHs) in Swedish smoked meat and fish. J. Food Compos. Anal..

[7] Lee S.Y., Lee J.Y., Shin H.S. (2015). Evaluation of Chemical Analysis Method and Determination of Polycyclic Aromatic Hydrocarbons Content from Seafood and Dairy Products. Toxicol. Res..

[8] International Agency for Research on Cancer (2005). Some Non-Heterocyclic Polycyclic Aromatic Hydrocarbons and Some Related Exposures.

[9] Cui Z., Ge N., Zhang A., Liu Y., Zhang J., Cao Y. (2015). Comprehensive determination of polycyclic aromatic hydrocarbons in Chinese herbal medicines by solid phase extraction and gas chromatography coupled to tandem mass spectrometry. Anal. Bioanal. Chem..

[10] Cai C., Chang G., Zhao M., Wu P., Hu Z., Jiang D. (2022). Determination of Polycyclic Aromatic Hydrocarbons in Traditional Chinese Medicine Raw Material, Extracts, and Health Food Products. Molecules.

[11] CRE (2015). Commission Regulation (EU) 2015/1933 of 27 October 2015 Amending Regulation (EC) No 1881/2006 as Regards Maximum Levels for Polycyclic Aromatic Hydrocarbons in Cocoa Fibre, Banana Chips, food Supplements, Dried Herbs and Dried Spices.

[12] Ministry of Food and Drug Safety (2013). Criteria and Method of Benzopyrene for Herbal in the Korean Herbal Pharmacopoeia.

[13] Chang K.-F., Fang G.-C., Chen J.-C., Wu Y.-S. (2006). Atmospheric polycyclic aromatic hydrocarbons (PAHs) in Asia: A review from 1999 to 2004. Environ. Pollut..

[14] Haleyur N., Shahsavari E., Mansur A.A., Koshlaf E., Morrison P.D., Osborn A.M., Ball A.S. (2016). Comparison of rapid solvent extraction systems for the GC–MS/MS characterization of polycyclic aromatic hydrocarbons in aged, contaminated soil. MethodsX.

[15] Shang D., Kim M., Haberl M. (2014). Rapid and sensitive method for the determination of polycyclic aromatic hydrocarbons in soils using pseudo multiple reaction monitoring gas chromatography/tandem mass spectrometry. J. Chromatogr. A.

[16] Humbert K., Debret M., Morin C., Cosme J., Portet-Koltalo F. (2022). Direct thermal desorption-gas chromatography-tandem mass spectrometry versus microwave assisted extraction and GC-MS for the simultaneous analysis of polyaromatic hydrocarbons (PAHs, PCBs) from sediments. Talanta.

[17] Paraíba L.C., Queiroz S.C., de Souza D.R., Saito M. (2011). Risk Simulation of Soil Contamination by Polycyclic Aromatic Hydrocarbons from Sewage Sludge used as Fertilizers. Soc. Bras. Química.

[18] Lingli Q., Wang S., Yang X., Sun C. (2019). MXene/reduced graphene oxide hydrogel film extraction combined with gas chromatography–tandem mass spectrometry for the determination of 16 polycyclic aromatic hydrocarbons in river and tap water. J. Chromatogr. A.

[19] Barco-Bonilla N., Romero-González R., Plaza-Bolaños P., Fernández-Moreno J.L., Garrido Frenich A., Martínez Vidal J.L. (2011). Comprehensive analysis of polycyclic aromatic hydrocarbons in wastewater using stir bar sorptive extraction and gas chromatography coupled to tandem mass spectrometry. Anal. Chim. Acta.

[20] Fernández-González V., Concha-Graña E., Muniategui-Lorenzo S., López-Mahía P., Prada-Rodríguez D. (2007). Solid-phase microextraction–gas chromatographic–tandem mass spectrometric analysis of polycyclic aromatic hydrocarbons: Towards the European Union water directive 2006/0129 EC. J. Chromatogr. A.

[21] Guillén M.D., Sopelana P., Partearroyo M.A. (2000). Determination of Polycyclic Aromatic Hydrocarbons in Commercial Liquid Smoke Flavorings of Different Compositions by Gas Chromatography−Mass Spectrometry. J. Agric. Food Chem..

[22] Moldoveanu S.C., Marshall J.W., Poole T.H. (2019). Extraction from Moist Snuff with Artificial Saliva of Benzo[a]pyrene and Other Polycyclic Aromatic Hydrocarbons. Contrib. Tob. Nicotine Res..

[23] Wang S.-W., Hsu K.-H., Huang S.-C., Tseng S.-H., Wang D.-Y., Cheng H.-F. (2019). Determination of polycyclic aromatic hydrocarbons (PAHs) in cosmetic products by gas chromatography-tandem mass spectrometry. J. Food Drug Anal..

[24] Serpe F.P., Esposito M., Gallo P., Serpe L. (2010). Optimisation and validation of an HPLC method for determination of polycyclic aromatic hydrocarbons (PAHs) in mussels. Food Chem..

[25] Shi L.-K., Zhang D.-D., Liu Y.-L. (2016). Survey of polycyclic aromatic hydrocarbons of vegetable oils and oilseeds by GC-MS in China. Food Addit. Contam. Part A.

[26] White S.F., Fernandes A., Rose M., Holland J., Walton P., Olivier L. (2004). Survey for Polycyclic Aromatic Hydrocarbons (PAHs) in Infant Formulae and Baby Foods.

[27] Tsutsumi T., Adachi R., Matsuda R., Watanabe T., Teshima R., Akiyama H. (2019). Concentrations of Polycyclic Aromatic Hydrocarbons in Smoked Foods in Japan. J. Food Prot..

[28] Varlet V., Serot T., Fabrice M., Le Bizec B., Prost (2007). C. Determination of PAH profiles by GC-MS/MS in salmon muscle meat processed with four cold smoking techniques. Food Addit. Contam..

[29] Johnson Y.S. (2012). Determination of Polycyclic Aromatic Hydrocarbons in Edible Seafood by QuEChERS-Based Extraction and Gas Chromatography-Tandem Mass Spectrometry. J. Food Sci..

[30] Yu L., Cao Y., Zhang J., Cui Z., Sun H. (2012). Isotope dilution-GC-MS/MS analysis of 16 polycyclic aromatic hydrocarbons in selected medicinal herbs used as health food additives. Food Addit. Contam. Part A Chem. Anal. Control Expo. Risk Assess..

[31] Szternfeld P., Marchi J., Malysheva S.V., Joly L. (2019). Modular Method for the Determination of Polycyclic Aromatic Hydrocarbons in Spices and Dried Herbs by Gas Chromatography–Tandem Mass Spectrometry. Food Anal. Methods.

[32] Hwang H.J., Lee S.H., Kim Y.Y., Shin H.S. (2021). Polycyclic Aromatic Hydrocarbon Risk Assessment and Analytical Methods Using QuEchERS Pretreatment for the Evaluation of Herbal Medicine Ingredients in Korea. Foods.

[33] Zhou D.-B., Han F., Ding L., Song W., Lv Y.-N., Hu Y.-Y., Liu Y.-X., Sheng X., Zheng P. (2020). Magnetic C60 nanospheres based solid-phase extraction coupled with isotope dilution gas chromatography–mass spectrometry method for the determination of sixteen polycyclic aromatic hydrocarbons in Chinese herbal medicines. J. Chromatogr. B.

[34] Anderson K.A., Szelewski M.J., Wilson G., Quimby B.D., Hoffman P.D. (2015). Modified ion source triple quadrupole mass spectrometer gas chromatograph for polycyclic aromatic hydrocarbon analyses. J. Chromatogr. A.

[35] Matuszewski B.K., Constanzer M.L., Chavez-Eng C.M. (2003). Strategies for the Assessment of Matrix Effect in Quantitative Bioanalytical Methods Based on HPLC−MS/MS. Anal. Chem..

[36] López-Fernández O., Rial-Otero R., González-Barreiro C., Simal-Gándara J. (2012). Surveillance of fungicidal dithiocarbamate residues in fruits and vegetables. Food Chem..

[37] Jeong S.H., Choi E.Y., Kim J., Lee C., Kang J., Cho S., Ko K.Y. (2021). LC-ESI-MS/MS Simultaneous Analysis Method Coupled with Cation-Exchange Solid-Phase Extraction for Determination of Pyrrolizidine Alkaloids on Five Kinds of Herbal Medicines. J. AOAC Int..

[38] (2016). Appendix F: Guidelines for Standard Method Performance Requirements. http://www.eoma.aoac.org/app_f.pdf.

[39] Ministry of Food and Drug Safety (2021). A Study on Method Improvement for the Safety Control of Herbal Medicine.

[40] Food Standards Agency, UK (2015). Interpretation of Margins of Exposure for Genotoxic Carcinogens.

