# Supplementary material for: GC-MS/MS Method for Determination of Polycyclic Aromatic Hydrocarbons in Herbal Medicines
Source: Molecules. 2023 May 2;28(9):3853. doi: 10.3390/molecules28093853 (PMC10179879; doi:10.3390/molecules28093853)
Supplement: Supplementary file 1 [file molecules-28-03853-s001.zip › molecules-2379477-supplementary.pdf]

Table S1. Matrix effects of 35 kinds of H.M.s.

No.	Name of herbal medicines	Matrix effects (%)							
		BaA [1]	Chry	BbF	BkF	BaP	IcdP	DahA	BghiP
1	Sanguisorbae Radix Carbonisatum	129	119	109	114	101	189	167	122
2	Aconiti Ciliare Tuber Preparata	136	122	114	119	109	152	165	130
3	Morindae Radix Preparata cum Vinum	131	119	116	119	107	144	153	124
4	Puerariae Radix	105	99	102	97	99	103	106	103
5	Glycyrrhizae Radix et Rhizoma	95	88	88	85	86	99	98	97
6	Platycodonis Radix	108	102	118	109	114	108	110	107
7	Angelicae Gigantis Radix	106	94	99	98	97	103	106	106
8	Atractylodis Rhizoma Alba	123	116	125	116	121	123	125	123
9	Bupleuri Radix	101	96	105	98	100	105	107	104
10	Achyranthis Radix	113	109	132	124	126	112	114	112
11	Ginseng Radix	107	103	110	102	106	111	113	110
12	Paeoniae Radix	93	89	96	88	92	94	95	94
13	Cnidii Rhizoma	97	93	97	89	94	100	101	99
14	Cyperi Rhizoma	96	94	108	99	91	94	102	99
15	Scutellariae Radix	112	105	124	116	121	112	114	109
16	Astragali Radix	107	103	129	116	122	108	109	107
17	Osterici seu Notopterygii Radix et Rhizoma	107	101	95	93	96	111	110	111
18	Zingiberis Rhizoma	97	95	91	85	92	106	103	101
19	Araliae Continentalis Radix	112	112	100	100	101	121	120	117
20	Liriopis seu Ophiopogonis Tuber	125	81	79	90	93	115	104	70
21	Aucklandiae Radix	97	86	81	79	81	99	94	88
22	Pinelliae Tuber	111	97	91	89	89	112	108	102
23	Saposhnikoviae Radix	113	99	97	93	97	116	110	89
24	Angelicae Dahuricae Radix	105	97	94	90	94	111	107	102
25	Aconiti Lateralis Radix Preparata	110	107	91	92	94	111	113	107
26	Dioscoreae Rhizoma	117	102	98	96	97	118	114	107
27	Acori Graminei Rhizoma	115	110	114	107	109	111	112	115
28	Asiasari Radix et Rhizoma	120	110	96	100	98	110	110	106
29	Linderae Radix	113	75	136	70	86	69	102	97
30	Clematidis Radix	107	99	109	103	107	108	107	104
31	Anemarrhenae Rhizoma	117	111	116	107	112	116	115	113
32	Atractylodis Rhizoma	105	122	85	138	116	104	99	81
33	Gastrodiae Rhizoma	117	109	115	109	109	116	118	120
34	Asparagi Tuber	107	100	110	107	104	111	112	110
35	Corydalis Tuber	90	83	89	86	83	93	95	91

[1] BaA: benzo[a]anthracene, Chry: chrysene, Bbf: benzo[b]fluoranthene, Bkf: benzo[k]fluoranthene, Bap: benzo[a]pyrene, IcdP: indeno[1,2,3-c,d]pyrene, DahA: dibenzo[a,h]anthracene, BghiP: benzo[g,h,i]perylene.

Table S2. LOD, LOQ, recoveries, intra-day and inter-day RSDs and matrix effects obtained using the GC-MS/MS in Scutellariae Radix.

No.	PAHs [1]	LOD (μg/kg)	LOQ (μg/kg)	Intra-day, n=9 [2]						Inter-day, n=9						Matrix Effect (%)
				Recovery (%)			RSD [3] (%)			Recovery (%)			RSDr [4] (%)			
				Low Level	Medium Level	High Level	Low Level	Medium Level	High Level	Low Level	Medium Level	High Level	Low Level	Medium Level	High Level	
1	BaA	0.19	0.58	125.0	98.8	78.1	5.6	2.7	1.7	111.4	88.4	69.1	12.0	11.6	11.3	112
2	Chry	0.23	0.69	107.5	66.2	54.7	6.6	6.9	2.2	89.5	64.9	52.8	32.1	11.4	3.1	105
3	BbF	0.24	0.71	121.5	107.9	86.6	7.2	5.1	2.0	122.3	96.8	75.2	6.7	12.7	13.2	124
4	BkF	0.14	0.43	101.3	71.4	60.1	9.4	6.2	1.0	76.2	61.7	51.7	30.5	13.8	14.2	116
5	BaP	0.37	1.11	105.9	87.3	71.4	6.2	7.0	0.5	99.9	77.3	62.2	10.9	11.9	13.0	121
6	IcdP	0.14	0.44	103.1	89.1	74.1	5.7	5.9	0.4	108.0	83.2	66.1	9.5	9.0	10.5	112
7	DahA	0.19	0.58	76.9	91.5	68.6	6.9	3.1	1.9	92.5	77.3	57.8	22.4	19.0	16.6	114
8	BghiP	0.36	1.1	113.6	84.3	67.8	5.7	12.3	0.7	111.3	81.6	63.3	5.3	8.3	8.2	109

[1] BaA: benzo[a]anthracene, Chry: chrysene, Bbf: benzo[b]fluoranthene, Bkf: benzo[k]fluoranthene, Bap: benzo[a]pyrene, IcdP: indeno[1,2,3-c,d]pyrene, DahA: dibenzo[a,h]anthracene, BghiP: benzo[g,h,i]perylene. [2] n: the number of analyses. [3] RSD: intra-day relative standard deviation (repeatability). [4] RSDr: inter-day relative standard deviation (reproducibility).

Table S3. Recoveries and precisions of 45 kinds of H.M.s for applicability excepting 6 representatives.

No.	Name of herbal medicines	BaA [1]		Chry		BbF		BkF		BaP		IcdP		DahA		BghiP	
		Recovery (%)	RSD (%)	Recovery (%)	RSD (%)	Recovery (%)	RSD (%)	Recovery (%)	RSD (%)	Recovery (%)	RSD (%)	Recovery (%)	RSD (%)	Recovery (%)	RSD (%)	Recovery (%)	RSD (%)
1	Glycyrrhizae Radix Preparata cum Mel	91.0	9.5	91.7	11.7	83.3	11.2	86.0	5.5	85.0	5.0	88.4	4.5	91.0	6.5	89.9	5.0
2	Glycyrrhizae Radix Preparata	83.9	9.5	89.0	14.4	77.3	10.0	77.6	5.6	89.7	13.9	78.2	4.4	80.6	4.2	80.1	8.1
3	Zingiberis Rhizoma Carbonisatum	109.9	11.8	97.8	9.7	114.5	17.7	78.2	9.4	115.9	13.9	107.1	4.0	96.7	3.5	107.4	5.6
4	Eucommiae Cortex Preparata cum Zingiberis Rhizoma Crudus	109.5	12.3	84.5	16.1	115.4	5.0	104.2	6.6	108.8	4.1	100.5	7.8	102.5	4.2	111.6	4.1
5	Eucommiae Cortex Preparata cum Sal	72.1	0.5	70.0	3.9	65.2	1.0	70.5	1.1	69.5	1.5	74.8	3.4	79.5	1.3	76.1	3.8
6	Eucommiae Cortex Carbonisatum	81.9	0.8	80.5	4.3	69.6	2.5	73.5	2.3	58.4	1.3	79.9	1.8	81.9	2.9	74.3	1.0
7	Rhei Rhizoma Preparata cum Vinum	90.8	8.8	87.0	12.1	91.9	6.0	87.2	11.1	116.4	19.7	85.3	12.3	84.2	10.9	88.3	7.4
8	Pinelliae Tuber cum Zingiberis Rhizoma Crudus et Alumen	84.0	8.1	81.4	11.3	93.2	4.1	79.3	1.8	95.0	19.8	81.6	2.1	69.2	2.6	87.7	3.3
9	Psoraleae Semen	82.0	4.4	69.4	4.6	69.1	3.8	72.2	1.7	74.1	19.1	74.4	1.7	67.2	4.2	73.0	3.1

	Preparata cum Sal																
10	Evodiae Fructus Preparata cum Glycyrrhizae Radix	85.4	15.9	83.7	12.7	86.8	11.6	76.7	4.8	96.0	15.0	82.0	3.8	100.2	3.5	80.0	8.4
11	Polygalae Radix Preparata cum Glycyrrhizae Radix	71.9	6.5	89.1	3.5	66.5	5.8	69.6	6.4	109.9	6.9	110.4	5.4	93.2	6.9	83.4	2.6
12	Polygalae Radix Preparata cum Mel	103.9	4.4	99.1	4.4	101.0	1.8	98.6	2.1	106.8	14.9	108.2	3.2	107.1	4.6	101.1	0.3
13	Aconiti Ciliare Tuber Preparata	86.9	5.2	89.7	1.2	72.3	8.3	70.6	9.0	106.1	5.7	98.9	11.1	115.3	10.7	88.9	10.5
14	Morindae Radix Preparata cum Vinum	93.0	5.0	72.5	11.6	66.6	17.3	113.5	7.5	101.3	8.9	73.8	15.0	69.8	5.8	83.4	6.2
15	Schizonepetae Spica Carbonisatum	91.0	11.1	82.0	19.5	89.5	9.7	96.3	5.8	100.6	4.6	104.7	3.7	101.6	2.1	117.9	8.2
16	Coptidis Rhizoma Preparata cum Vinum	95.4	14.6	97.5	14.1	98.5	14.3	90.6	9.0	87.8	11.4	89.9	2.6	88.6	0.1	88.7	13.1
17	Phellodendri Cortex Preparata cum Sal	113.5	10.5	99.6	11.0	103.9	10.3	105.3	7.5	80.0	14.7	108.5	7.6	105.6	6.4	105.5	6.9
18	Siegesbeckiae Herba Preparata cum Vinum	100.5	3.4	99.1	2.9	78.3	1.9	88.4	1.5	51.5	5.9	103.7	1.1	94.1	2.9	95.0	1.5

19	Puerariae Radix	83.7	1.9	63.9	1.7	78.0	1.7	63.6	1.3	85.5	1.9	110.7	2.6	120.2	2.2	79.6	1.0
20	Glycyrrhizae Radix et Rhizoma	94.6	5.3	82.0	2.9	80.0	4.3	65.9	2.4	91.4	0.6	110.0	2.3	116.9	1.3	71.2	3.3
21	Platycodonis Radix	73.2	6.0	60.5	7.6	71.0	4.2	67.3	2.4	72.3	6.7	73.1	1.5	72.2	5.8	85.0	10.1
22	Atractylodis Rhizoma Alba	75.5	9.4	57.5	9.4	66.0	11.7	59.2	11.7	62.6	20.9	68.3	6.9	61.6	6.3	62.4	4.3
23	Bupleuri Radix	86.2	2.6	69.5	0.4	79.3	1.5	73.5	2.7	80.8	5.4	82.7	4.5	73.2	3.2	82.7	2.4
24	Ginseng Radix	83.0	2.7	74.9	0.4	81.3	1.5	83.4	2.9	85.8	5.4	75.4	4.5	70.2	3.2	81.4	2.4
25	Paeoniae Radix	79.4	16.8	79.2	20.3	88.4	21.1	80.0	16.5	76.8	24.9	73.0	13.3	71.2	11.4	81.8	15.8
26	Cnidii Rhizoma	77.8	2.3	69.5	3.0	78.5	3.4	71.9	5.0	74.7	2.2	74.4	1.8	68.2	4.5	77.2	10.0
27	Cyperi Rhizoma	78.8	3.9	73.1	3.9	81.1	2.9	87.1	2.1	83.4	8.9	78.1	4.3	75.1	3.5	83.4	3.7
28	Astragali Radix	71.4	6.1	63.6	8.0	69.2	6.6	72.1	9.9	63.6	17.5	64.9	5.2	108.5	1.6	76.5	5.5
29	Osterici seu Notopterygii Radix et Rhizoma	75.2	10.5	66.7	23.9	84.8	2.7	60.9	10.4	55.1	18.8	67.7	7.1	64.9	11.7	68.7	12.3
30	Zingiberis Rhizoma	105.5	7.0	82.2	9.7	73.2	7.0	71.6	7.1	96.2	54.9	92.1	6.5	89.8	7.1	89.7	4.4
31	Araliae Continentalis Radix	105.3	13.3	99.6	11.8	70.5	12.3	72.4	13.4	78.3	15.9	99.1	11.9	90.5	13.7	88.0	14.7
32	Liriopis seu Ophiopogonis Tuber	104.6	5.4	94.1	5.8	82.9	5.1	82.0	9.4	110.3	5.6	104.6	8.9	92.4	6.4	100.6	13.7
33	Aucklandiae Radix	114.6	5.3	96.8	10.4	79.2	8.3	84.6	4.1	78.6	3.6	84.9	4.8	73.4	7.1	90.6	4.0
34	Pinelliae Tuber	103.5	10.1	96.5	13.3	74.9	8.4	77.2	8.7	77.8	12.2	101.4	13.3	95.9	11.0	92.6	15.2
35	Saposhnikoviae Radix	87.3	5.3	85.8	2.5	86.8	3.3	88.1	3.3	116.1	25.3	84.4	4.4	75.2	3.5	89.4	3.4

36	Angelicae Dahuricae Radix	112.6	8.0	94.9	17.6	84.7	17.5	86.8	15.8	90.3	24.6	112.7	15.3	104.9	12.5	94.5	20.0
37	Aconiti Lateralis Radix Preparata	113.5	2.5	101.5	0.4	90.8	5.4	95.0	8.9	90.1	26.4	108.7	4.3	104.8	3.3	101.8	9.0
38	Dioscoreae Rhizoma	93.1	7.9	86.4	8.5	90.3	10.5	97.6	13.3	51.1	27.4	75.5	5.9	88.3	24.3	70.6	14.1
39	Acori Graminei Rhizoma	116.6	25.0	118.1	33.6	85.6	22.7	84.9	13.1	105.8	31.7	91.6	15.4	76.5	7.3	76.8	23.7
40	Linderae Radix	109.6	22.0	83.6	29.9	101.9	27.9	70.9	25.3	85.5	24.1	82.1	22.2	81.9	24.3	89.4	23.4
41	Clematidis Radix	126.1	4.9	88.0	10.2	119.0	4.9	92.1	6.8	107.2	23.5	95.4	10.6	76.7	21.8	121.7	4.7
42	Atractylodis Rhizoma	122.3	5.7	93.3	7.5	110.5	8.3	89.3	2.4	100.9	5.8	111.2	5.3	104.6	4.2	106.4	6.3
43	Gastrodiae Rhizoma	99.4	5.0	94.6	5.0	88.9	3.2	79.0	4.6	89.0	2.9	96.9	1.7	98.8	2.6	94.5	0.9
44	Asparagi Tuber	74.2	6.4	64.7	6.0	65.5	15.8	63.6	11.7	67.9	6.3	63.9	7.6	74.9	9.8	67.3	16.0
45	Corydalis Tuber	75.2	13.6	54.2	22.3	94.6	6.7	67.4	7.1	65.3	13.4	91.6	12.9	102.7	16.5	115.9	21.4

[1] BaA: benzo[a]anthracene, Chry: chrysene, Bbf: benzo[b]fluoranthene, Bkf: benzo[k]fluoranthene, Bap: benzo[a]pyrene, IcdP: indeno[1,2,3-c,d]pyrene, DahA: dibenzo[a,h]anthracene, BghiP: benzo[g,h,i]perylene.

Table S4. The item lists of the sum of 8 PAHs is over 50 μg/kg among 459 samples of 51 kinds of herbal medicines and maximum concentration of each PAH in the same items.

No.	Name of H.M.s	$N_{over}(N_{sam})$ [1]	Maximum Concentration (μg/kg)							
			BaA [2]	CHR	BbF	BkF	BaP	IcdP	DahA	BghiP
1	Puerariae Radix	1(13)	9.74	13.53	11.01	4.25	2.09	4.81	1.48	4.34
2	Glycyrrhizae Radix Preparata	1(9)	4.43	36.74	19.75	16.65	0.00	0.00	0.00	0.00
3	Osterici seu Notopterygii Radix et Rhizoma	1(10)	12.64	12.64	12.64	12.64	12.64	12.64	12.64	12.64
4	Zingiberis Rhizoma	1(9)	25.82	94.88	47.26	13.35	38.29	18.00	0.00	33.34
5	Zingiberis Rhizoma Carbonisatum	1(5)	6.09	40.66	22.88	6.20	3.65	1.49	0.00	1.25
6	Platycodonis Radix	2(12)	13.09	18.14	13.09	5.15	2.46	6.01	1.38	4.02
7	Rhei Rhizoma Preparata cum Vinum	3(7)	58.50	55.32	25.82	7.44	10.59	5.72	1.14	4.86
8	Aucklandiae Radix	2(2)	24.12	122.87	19.29	8.12	12.47	4.00	0.00	4.99
9	Pinelliae Tuber	2(10)	15.75	42.00	12.65	2.49	2.52	1.12	1.11	1.11
10	Pinelliae Tuber cum Zingiberis Rhizoma Crudus et Alumen	1(9)	16.63	23.27	13.58	1.78	1.59	1.61	1.02	0.00
11	Clematidis Radix	2(9)	8.39	35.93	13.12	3.12	6.77	2.64	1.12	3.96
12	Gastrodiae Rhizoma	1(10)	42.13	94.17	27.46	8.52	16.63	3.26	0.00	3.75
13	Morindae Radix Preparata cum Vinum	1(6)	25.37	32.13	15.62	3.77	8.22	3.73	3.13	2.97
14	Corydalis Tuber	2(10)	13.75	55.07	12.30	2.29	7.16	2.16	0.00	4.09
15	Schizonepetae Spica Carbonisatum	1(4)	5.38	20.00	15.87	3.64	4.37	8.42	1.53	6.47
16	Scutellariae Radix	2(12)	30.06	36.21	32.81	9.55	25.70	12.19	2.06	38.48
17	Coptidis Rhizoma Preparata cum Vinum	5(5)	38.09	42.49	26.42	7.19	17.22	11.73	1.74	9.62
18	Phellodendri Cortex Preparata cum Sal	5(8)	25.26	87.89	52.95	4.29	6.78	5.67	4.81	5.12
19	Siegesbeckiae Herba Preparata cum Vinum	2(4)	15.27	83.17	29.56	6.19	4.60	9.83	2.10	10.79

[1] N_{over}: the number of samples in which the sum of 8 PAHs is greater than 50μg/kg, N_{sam}: the number of samples examined. [2] BaA: benzo[a]anthracene, Chry: chrysene, Bbf: benzo[b]fluoranthene, Bkf: benzo[k]fluoranthene, Bap: benzo[a]pyrene, IcdP: indeno[1,2,3-c,d]pyrene, DahA: dibenzo[a,h]anthracene, BghiP: benzo[g,h,i]perylene.
